# 15.3% Efficiency All‐Small‐Molecule Organic Solar Cells Achieved by a Locally Asymmetric F, Cl Disubstitution Strategy

**DOI:** 10.1002/advs.202004262

**Published:** 2021-02-22

**Authors:** Dingqin Hu, Qianguang Yang, Yujie Zheng, Hua Tang, Sein Chung, Ranbir Singh, Jie Lv, Jiehao Fu, Zhipeng Kan, Bo Qin, Qianqian Chen, Zhihui Liao, Haiyan Chen, Zeyun Xiao, Kuan Sun, Shirong Lu

**Affiliations:** ^1^ Chongqing Institute of Green and Intelligent Technology Chongqing School University of Chinese Academy of Sciences (UCAS Chongqing) Chinese Academy of Sciences Chongqing 400714 China; ^2^ Key Laboratory of Low‐Grade Energy Utilization Technologies and Systems (Ministry of Education) School of Energy and Power Engineering Chongqing University Chongqing 400044 P. R. China; ^3^ Chongqing School University of Chinese Academy of Sciences Chongqing 400714 China; ^4^ Department of Chemical Engineering Pohang University of Science and Technology Pohang Pohang 790‐784 South Korea; ^5^ Department of Energy and Materials Engineering Dongguk University Seoul 100–715 Republic of Korea

**Keywords:** organic solar cells, all small molecule, asymmetric disubstitution strategy

## Abstract

Single junction binary all‐small‐molecule (ASM) organic solar cells (OSCs) with power conversion efficiency (PCE) beyond 14% are achieved by using non‐fullerene acceptor Y6 as the electron acceptor, but still lag behind that of polymer OSCs. Herein, an asymmetric Y6‐like acceptor, BTP‐FCl‐FCl, is designed and synthesized to match the recently reported high performance small molecule donor BTR‐Cl, and a record efficiency of 15.3% for single‐junction binary ASM OSCs is achieved. BTP‐FCl‐FCl features a F,Cl disubstitution on the same end group affording locally asymmetric structures, and so has a lower total dipole moment, larger average electronic static potential, and lower distribution disorder than those of the globally asymmetric isomer BTP‐2F‐2Cl, resulting in improved charge generation and extraction. In addition, BTP‐FCl‐FCl based active layer presents more favorable domain size and finer phase separation contributing to the faster charge extraction, longer charge carrier lifetime, and much lower recombination rate. Therefore, compared with BTP‐2F‐2Cl, BTP‐FCl‐FCl based devices provide better performance with FF enhanced from 71.41% to 75.36% and *J*_sc_ increased from 22.35 to 24.58 mA cm^−2^, leading to a higher PCE of 15.3%. The locally asymmetric F, Cl disubstitution on the same end group is a new strategy to achieve high performance ASM OSCs.

## Graphical abstract

Single‐junction all‐small‐molecule organic solar cells with record high PCEs of 15.3% have been demonstrated by a locally asymmetric electron acceptor BTP‐FCl‐FCl and BTR‐Cl donor.

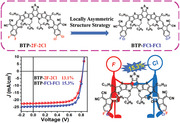

## 1 Introduction

Solution‐processed bulk‐heterojunction (BHJ) OSCs have a promising application prospect in flexible and portable devices by virtue of tunable absorption range, low material cost, potential for large area device fabrication, and light‐weight.^[^ ^1^, ^2^, ^3^, ^4^ ^]^ ASM OSC as a special kind of BHJ OSC, has arisen as a more preferred candidate due to the well‐defined structure and high purity of the small molecules, and the high reproducibility of the devices.^[^ ^5^, ^6^, ^7^, ^8^ ^]^ Recently, non‐fullerene acceptors (NFAs) based on Y6 have attracted extensive attentions and experienced a rapid progress in PCE.^[^ ^9^, ^10^, ^11^, ^12^ ^]^ Employing the Y6 based NFA, single‐junction binary ASM OSCs with above 14% PCE and ternary ASM OSCs with above 15.8% PCE have been reported.^[^ ^13^, ^14^, ^15^, ^16^ ^]^ However, the performance of ASM OSCs still lags behind the polymer donor based OSCs which show PCEs exceeding 18% in single junction binary devices.^[^ ^17^ ^]^ The main reason underlying the inferior PCEs of ASM OSCs is that it is difficult to control the morphology of the ASM based active layer that limits the charge separation and transport process. Considering the advantages of small molecules, it is clearly necessary to further improve the photovoltaic performance of ASM OSCs.

To further improve the performance of Y6 and to investigate the structure property relationship, a few studies modifying the Y6 structure have been reported. For example, changing the type and number of the halogen element (F, Cl, and Br) on the end group, introducing different end groups into the core skeleton to construct asymmetric structures, changing the side chain length of the conjugated skeleton to adjust the energy level, crystallinity, and solubility, etc.^[^ ^18^, ^19^, ^20^, ^21^, ^22^ ^]^ As such, F atoms on the Y6 end group were replaced with Cl atoms to synthesize BTP‐4Cl and the OSC PCEs increased from 15.6% to 16.5%.^[^ ^23^ ^]^ IC‐2F and IC‐2Cl end groups were introduced to the BTP core to synthesize an asymmetric molecule BTP‐2F‐2Cl affording 16.83% PCE in polymer OSCs.^[^ ^24^ ^]^ It is worth noting that the photovoltaic performance of OSCs enhanced with BTP‐4Cl or BTP‐2F‐2Cl acceptor when polymer donors were used, however BTP‐4Cl and BTP‐2F‐2Cl do not match well with strong crystalline small molecule donor materials such as BTR‐Cl. Thus, to design new Y6 based acceptor materials to attain appropriate crystallinity and phase separation with small molecule donor for ASM OSCs is of great importance.

Energy level alignment (ELA) is an important indicator for designing new OSC materials, and especially useful when predicting the device performance potential consisting of materials with similar energy levels.^[^ ^25^, ^26^ ^]^ Recently, the electronic static potential (ESP) distributions of non‐fullerene and fullerene acceptors were compared in literature.^[^ ^26^ ^]^ Most of the them exhibit a positive ESP, and the value for IT‐4F is larger than that of PC_71_BM. Moreover, most of the donor materials have a negative ESP. Therefore, the local ESP difference between the donor and acceptor at the D–A interface will generate an intermolecular electric field (IEF), which will facilitate (or hamper for some cases) exciton dissociation. Thus, attaining molecules with suitable ESP is necessary to achieve high‐efficiency OSCs. Random disubstitution of the end groups of a small molecule acceptor has shown the potential of adjusting the ESP and providing appropriate ELA.^[^ ^27^, ^28^ ^]^

Compared with the globally asymmetric isomer BTP‐2F‐2Cl, the average ESP of locally asymmetric F, Cl disubstitution BTP‐FCl‐FCl is larger and the ESP distribution is less disorder, resulting in stronger and more orderly IEF formed between BTR‐Cl donor and BTP‐FCl‐FCl acceptor, which is beneficial for charge generation and extraction. In addition, BTP‐FCl‐FCl reveals appropriate crystallinity, more favorable domain size, and phase separation. In comparison with the BTP‐2F‐2Cl based devices, the BTP‐FCl‐FCl based devices show faster charge extraction, longer charge carrier lifetime, and additionally, much less recombination. Therefore, optimized FF and *J*_SC_ are improved from 71.41% to 75.36% and 22.35 to 24.58 mA cm^−2^, with *V*_OC_ maintained, leading to a higher PCE of 15.3%.

## 2 Results and Discussions

### 2.1 Synthesis and Photoelectrical Properties

As shown in Scheme S1, Supporting Information, a locally asymmetric acceptor BTP‐FCl‐FCl was synthesized via Knoevenagel condensation reactions between the dialdehyde compound 1 (BT‐CHO, Scheme S1, Supporting Information) and two different end groups. The chemical structure of BTP‐FCl‐FCl was fully characterized by ^1^H nuclear magnetic resonance (NMR), ^13^C NMR, ^19^F NMR, and Fourier‐transform mass spectrometry (FT‐MS). The overall ratio of F to Cl in BTP‐FCl‐FCl is 1:1 (Figure S7, Supporting Information). The new acceptor material show good thermal stability (Figure S1, Supporting Information) and good solubility in commonly used solvents, including chloroform (CF), dichloromethane (DCM), and chlorobenzene (CB).

**Figure 1:**
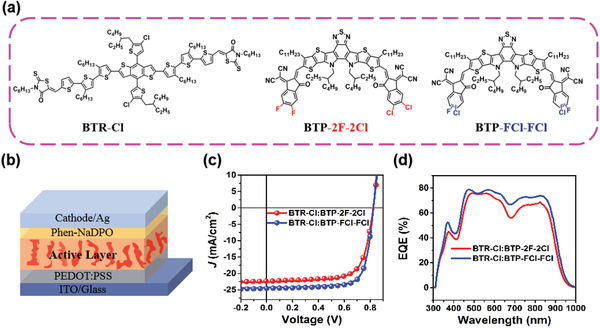
a) Chemical structures of BTP‐2F‐2Cl, BTP‐FCl‐FCl, and BTR‐Cl. b) Schematic illustration of the device structure employed in this work. c) Current density–voltage (*J*–*V*) curves. d) External quantum efficiency (EQE) of BTP‐2F‐2Cl, BTP‐FCl‐FCl based devices.

The chemical structures, energy levels, and ultraviolet–visible (UV–vis) absorption spectra of BTP‐2F‐2Cl and BTP‐FCl‐FCl are shown in **Figure** **1**, Figure S2 and Table S1, Supporting Information. As determined by cyclic voltammetry, the BTP‐FCl‐FCl exhibits lower highest occupied molecular orbital (HOMO) and lowest unoccupied molecular orbital (LUMO) energy levels compared with BTP‐2F‐2Cl, respectively (Figure S2b,c and Table S1, Supporting Information). The optical gaps (*E*_g_^opts^) of the two acceptor films are both 1.33 eV, which agree well with the energy levels estimated by cyclic voltammetry (Table S1, Supporting Information). As from the absorption spectra (Figure S2a and Table S1, Supporting Information), the main film absorption peak is located at 835.5 nm for BTP‐2F‐2Cl, and 845.5 nm for BTP‐FCl‐FCl, respectively. The reported efficient small molecule donor BTR‐Cl displays strong absorption at 450–700 nm, which is complementary with BTP‐FCl‐FCl acceptor in absorption and energy levels.

### 2.2 Photovoltaic Properties

Devices with conventional architecture of indium tin oxide (ITO)/poly(3,4‐ethylenedioxythiophene):poly(styrenesulfonate) (PEDOT:PSS)/active layer (BTR‐Cl: BTP‐2F‐2Cl, BTP‐FCl‐FCl)/Phen‐NaDPO/Ag (Figure 1b) were fabricated. The device fabrication conditions including donor/acceptor weight ratios, thermal annealing temperature, active layer thickness, and solvent annealing were carefully optimized. The corresponding data are summarized in Tables S2–S7, Supporting Information. The current density–voltage (*J*–*V*) of the best devices are plotted in Figure 1c and the detailed photovoltaic parameters are listed in **Table** **1**. As compared, the BTP‐FCl‐FCl based device presents excellent device performance, with a *V*_oc_ of 825.4 mV, a *J*_sc_ of 24.58 mA cm^−2^, an FF of 75.36%, and a PCE of 15.29% (Table 1). As for the BTP‐2F‐2Cl based device, a *V*_oc_ of 823.4 mV, a *J*_sc_ of 22.35 mA cm^−2^, an FF of 71.41%, and a PCE of 13.14% was obtained (Table 1). These results indicate that the locally asymmetric acceptor BTP‐FCl‐FCl with a F, Cl disubstituted end group shows better photovoltaic performance than that of the globally asymmetric acceptor BTP‐2F‐2Cl. The main contributor to the PCE enhancement is from the *J*_sc_ and FF increase and the detailed analysis is following.

**Table 1:** Photovoltaic performance of BTR‐Cl:acceptor based organic solar cells

Device condition	*V*_oc_ [mV]	*J*_sc_ [mA cm^−2^]	FF [%]	PCE^a)^ [%]	*J*_sc_ ^b)^ [mA cm^−2^]
BTR‐Cl:BTP‐2F‐2Cl	823.4	22.35	71.41	13.1 (12.83 ± 0.29)	22.03
BTR‐Cl:BTP‐FCl‐FCl	825.4	24.58	75.36	15.3 (15.14 ± 0.09)	24.26

^a)^ Statistical data obtained from 15 devices

^b)^ The *J*_sc_ calculated from external quantum efficiency (EQE) curves.

Figure 1d shows the external quantum efficiency (EQE) spectra of the BTP‐2F‐2Cl and BTP‐FCl‐FCl based devices. The EQE curves of the two devices are very similar in shape, but the BTP‐FCl‐FCl based device exhibits a stronger photoresponse. In addition, the EQE curve of the BTP‐FCl‐FCl based device red‐shifts about 10 nm which is consistent with the blend film absorption (Figure S3, Supporting Information). These two factors contribute to the increased *J*_sc_ of the BTP‐FCl‐FCl based device. The *J*_sc_ values of the BTP‐2F‐2Cl and BTP‐FCl‐FCl based devices integrated from the EQE spectra are 22.03 and 24.26 mA cm^−2^, respectively, consistent with the *J*_sc_ values measured from the solar simulator (within a 1.5% error, Table 1).

**Figure 2:**
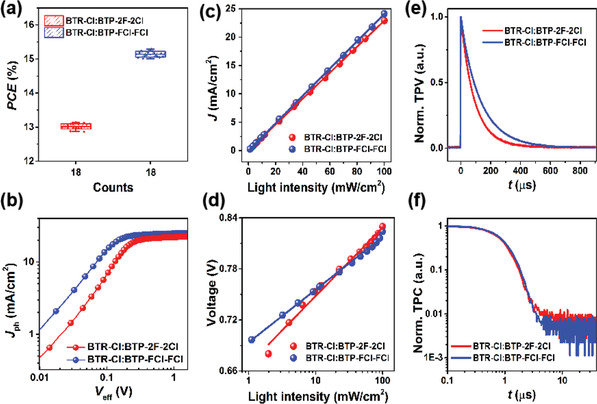
a) The PCE distribution, b) *J*_ph_ versus *V*_eff_ curves, c) *J*_sc_ and d) *V*_oc_ dependence on the light intensity, e) transient photovoltage, and f) transient photocurrent of BTP‐2F‐2Cl, BTP‐FCl‐FCl based devices.

As depicted from the distribution of PCE visualized in the statistical chart (**Figure** **2**a), the photovoltaic performance of the locally asymmetric acceptor BTP‐FCl‐FCl with a F, Cl disubstituted end group enhanced greatly. In addition to *J*_sc_, another prominent enhanced parameter is the FF, which is the most significant contributor to the performance enhancement. As previously reported, FF is largely correlated with the competition between charge recombination and extraction.^[^ ^29^ ^]^ In this work, we adopted a locally asymmetric structure strategy to enhance the FF significantly. It is necessary to analyze this key parameter via measuring the transportation, extraction, collection, and recombination behaviors of photo‐generated charges.

To investigate the exciton dissociation and extraction processes, the photocurrent density (*J*_ph_) as a function of the effective voltage (*V*_eff_) was plotted (Figure 2d) using the equation *J*_ph_ = *J*_L_−*J*_D_, in which *J*_L_ is the current density under illumination and *J*_D_ is that in the dark; *V*_eff_ = *V*_0_−*V*_A_, where *V*_0_ is the voltage when *J*_ph_ is equal to 0, and *V*_A_ is the applied bias voltage.^[^ ^30^ ^]^ Scanning voltage from −1.5 to 1.5 V, the gross photogenerated excitons are assumed to dissociate into free charge carriers and then collected by the electrodes. The exciton dissociation efficiency (*η*_diss_ = *J*_sc_ / *J*_sat_) and charge collection efficiency (*η*_coll_ = *J*_max power_ / *J*_sat_) were calculated under the short‐circuit and maximum power output conditions,^[^ ^31^ ^]^ and the corresponding data are listed in Table S9, Supporting Information. As expected, the BTP‐FCl‐FCl based device presents a *η*_diss_ of 97.02% and a *η*_coll_ of 88.26%, higher than BTP‐2F‐2Cl based device with a *η*_diss_ of 95.29% and a *η*_coll_ of 83.55%, indicating that the locally asymmetric structure strategy facilitates exciton dissociation as well as charge collection.

To measure the dynamics of free charges in the devices, *J*–*V* curves under various incident light intensities and the transient photovoltage/photocurrent (TPV/TPC) were recorded (Figure 2e,f). From literatures, the bimolecular recombination behavior could be quantified by employing the dependences of *V*_oc_ and *J*_sc_ on light intensity (*P*_light_).^[^ ^32^, ^33^ ^]^ In this sense, the relationship between *J*_sc_ and the light intensity (*P*_light_) was analyzed thereafter to evaluate the degree of bimolecular recombination in the devices. From Figure 2c, *α* values of 0.992 and 0.997 were obtained for the BTP‐2F‐2Cl, BTP‐FCl‐FCl based devices, respectively, which are indicative of negligible bimolecular recombination losses. In addition to bimolecular recombination losses, Shockley–Read–Hall (SRH) recombination is another key factor to explore.^[^ ^34^, ^35^ ^]^ In this case, *V*_oc_ was plotted as a function of the incident light intensity (Figure 2d). The data were fitted according to the expression *V*_oc_ ∝ *n*k*T*/*q* ln(*I*), where k is the Boltzmann constant, *T* is the Kelvin temperature, and *q* is the elementary charge, respectively. As depicted in Figure 2d, the *n* values of 1.34 and 1.03 for BTP‐2F‐2Cl, BTP‐FCl‐FCl based devices were obtained, indicating that the electrostatic potential control induced by Cl atom and F atom in the same end group generates less trap‐assisted recombination centers, which accounts for the photovoltaic performance improvement of the BTP‐FCl‐FCl based devices.

To investigate the charge extraction and recombination patterns of the two asymmetric acceptor based devices. We adopted TPV and TPC measurements to obtain the charge extraction time and carrier lifetime. The carrier lifetime (*τ*) under open‐circuit condition (Figure 2e) was extracted from the TPV decay dynamics employing mono‐exponential fits under dark condition. The fitting of the TPV signal with equation: *y* = A*exp(−*x* / *t*),^[^ ^36^ ^]^ and values of 75.8 and 131.6 µs were obtained for the BTP‐2F‐2Cl and BTP‐FCl‐FCl based devices, respectively. The *τ* of BTP‐FCl‐FCl based devices is ≈1.7 times longer than that of the BTP‐2F‐2Cl based devices, implying that the charge recombination is greatly suppressed in the device with the locally asymmetric acceptor, in line with the weaker recombination in BTP‐FCl‐FCl based devices. Meanwhile, the charge extraction time of the BTP‐2F‐2Cl and BTP‐FCl‐FCl based devices are 0.78 and 0.91 µs as depicted in TPC (Figure 2f), indicating that the charge extraction from the locally asymmetric acceptor is as efficient as that from the BTP‐2F‐2Cl based device. These results illustrate that this locally asymmetric structure strategy can effectively facilitate the charge carrier extraction and suppress recombination, which is in agreement with previous *J*_ph_–*V*_eff_ measurements.

The electron and hole mobilities were derived by fitting the dark current density with the space charge limited current model.^[^ ^37^ ^]^ To get the dark current density, the electron only and hole only devices with architectures of ITO/PEDOT:PSS/AL/MoO_3_/Ag and ITO/ZnO/Phen‐NaDPO/AL/Phen‐NaDPO/Ag were fabricated. The electron and hole mobility values are presented in Figure S5 and Table S8, Supporting Information. Specifically, the electron and hole mobility values for BTP‐FCl‐FCl based devices are 2.21×10^–3^, and 2.02 ×10^–3^ cm^2^ V^–1^ s^–1^. Notably, the slightly increased electron and hole mobilities afford more balanced electron and hole mobilities compared to BTP‐2F‐2Cl based devices. As a result, the more balanced charge mobilities (*μ*_e_ / *μ*_h_ = 1.09) results in the higher FF of 75.36% in the locally asymmetric acceptor system.

### 2.3 Blend Morphology Characterization

**Figure 3:**
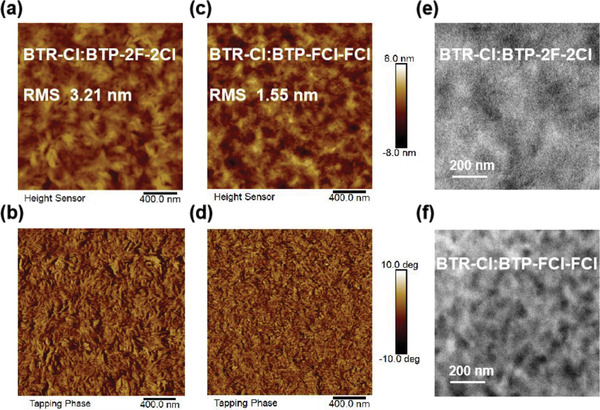
a–c) AFM height images (2 × 2 µm), b–d) AFM phase images (2 × 2 µm), and e–h) TEM images (scale bar 200 nm) of BTR‐Cl:BTP‐2F‐2Cl, BTR‐Cl:BTP‐FCl‐FCl blend films.

In order to explore the donor/acceptor phase separation, we carried out morphology characterizations of the blend films via atomic force microscopy (AFM) and transmission electron microscopy (TEM). As demonstrated in AFM height images (**Figure** **3**a,c), the BTP‐FCl‐FCl based blend film has a root mean square roughness (*R*_q_) value of 1.55 nm, which is smaller than that of BTP‐2F‐2Cl based film (*R*_q_ = 3.21 nm) implying that the most homogeneously distributed morphology formed therein. And from the AFM phase images (Figure 3b,d), the BTP‐FCl‐FCl based film presents a finer phase separation, which is beneficial for the charge dissociation because of the larger donor/acceptor interfacial area.^[^ ^38^ ^]^ As shown in the TEM images (Figure 3e,f), the BTP‐FCl‐FCl based blend film displays donor/acceptor interpenetrating networks and a pronounced and clear phase separation closer to the optimal domain size of 20 nm,^[^ ^39^ ^]^ as compared to the BTP‐2F‐2Cl based blend film. These morphology data are in accordance with the information from *J*_ph_–*V*_eff_ and mobility measurements.

**Figure 4:**
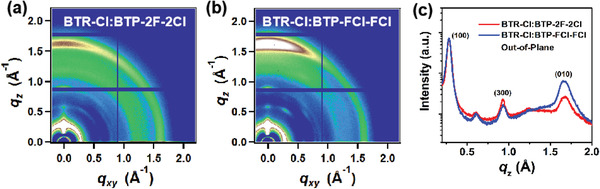
a,b) 2D GIWAXS patterns and c) out‐of‐plane line‐cut profiles of the BTP‐2F‐2Cl, BTP‐FCl‐FCl based blend films.

Grazing‐incidence wide‐angle X‐ray scattering (GIWAXS) studies were performed to investigate the molecular packing and orientation in the thin films, the 1D patterns out‐of‐plane (OOP) profiles of these films are presented in **Figure** **4** and Figure S6, Supporting Information, the corresponding GIWAXS parameters are summarized in Tables S10, Supporting Information. The BTR‐Cl film exhibits a preferential edge‐on orientation in agreement with previously reported work,^[^ ^40^, ^41^ ^]^ whereas the BTP‐2F‐2Cl and BTP‐FCl‐FCl films demonstrate a preferential face‐on orientation (Figure S6, Supporting Information). In addition, the BTP‐FCl‐FCl based blend film exhibits (010) *π*–*π* peaks at *q*_z_ = 1.67 Å^−1^ with a CCL of 45 Å, and BTP‐2F‐2Cl based blend film presents (010) *π*–*π* peaks at *q*_z_ = 1.67 Å^−1^ with a CCL of 48 Å. The identical molecule face‐on orientation and the smaller CCL of the *π*–*π* peak in the BTP‐FCl‐FCl based blend film further prove that the locally asymmetric acceptor affords finer phase separation with the BTR‐Cl donor,^[^ ^42^ ^]^ which is beneficial for charge separation, as well as the reduced charge recombination, and this trend is in agreement with AFM results.

### 2.4 Calculations

From experimental results, the BTP‐FCl‐FCl based devices present better photovoltaic performance than the BTP‐2F‐2Cl based devices. However, their HOMO and LUMO are nearly the same and could not provide more appropriate driving force for generating free charge by ELA. Recently, researchers proved that the intermolecular electric fields (IEF) produced by the ESP difference between the donor and accepter molecules can provide the driving force.^[^ ^25^, ^26^ ^]^ Therefore, we also analyzed the ESP distributions of BTP‐2F‐2Cl and BTP‐FCl‐FCl molecules.

The ESP *V(* ***r***) at any point ***r*** is defined as sum of the Coulomb potential of all ions and electrons^[^ ^26^, ^43^ ^]^

(1) $$V\left(r\right)=\frac{1}{4\pi \varepsilon_{0}}\left[\sum_{A}\frac{Z_{A}e}{\left|R_{A}-r\right|}-e\int \frac{\rho \left(r^{\prime }\right)dr^{\prime }}{r^{\prime }-r}\right]$$

where *ɛ_0_* is dielectric constant, *Z*_A_ is charge on ion A, *ρ(r)* is the charge density, and *e* is the electron charge. The relaxed atomic and electronic structures were obtained by DFT calculations at the B3LYP/6‐31G basis set, and all DFT calculations were carried out using Gaussian 09. The ESP were analyzed and visualized by Multiwfn^[^ ^44^ ^]^ and VMD,^[^ ^45^ ^]^ respectively.

**Figure 5:**
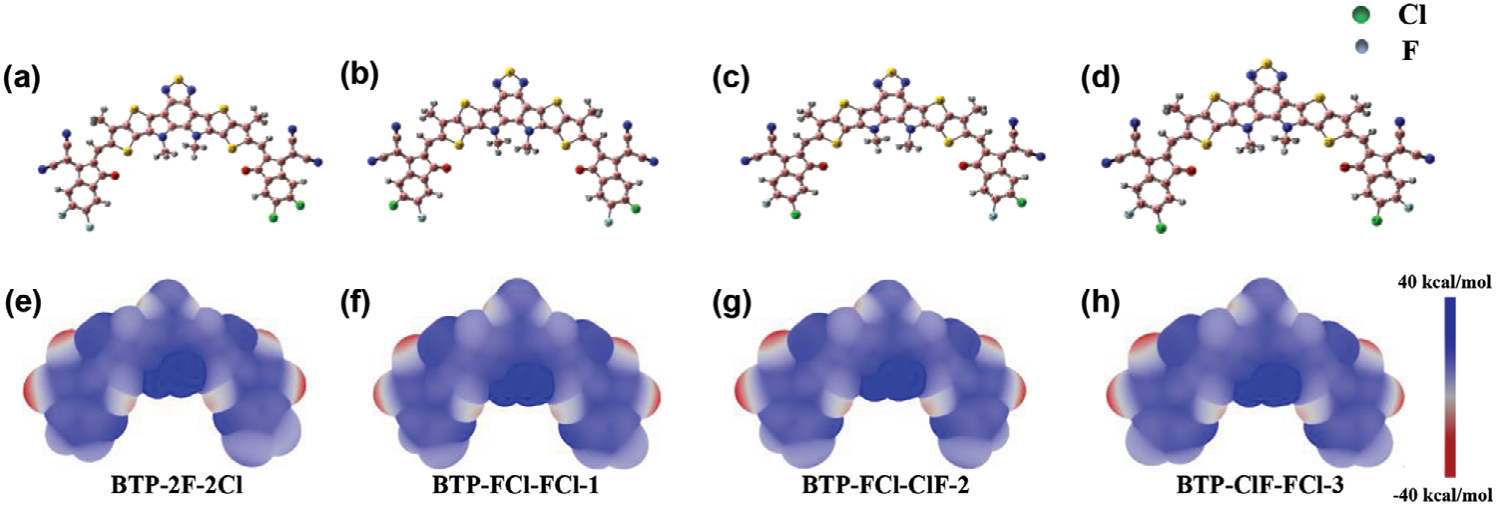
a–d) Molecular structures and ESP distributions of BTP‐2F‐2Cl, BTP‐FCl‐FCl‐1, BTP‐FCl‐FCl‐2, and BTP‐FCl‐FCl‐3, respectively. e–g) Averaged ESP values of the atoms around the halogen atoms of the BTP‐2F‐2Cl compared with BTP‐FCl‐FCl‐1, BTP‐FCl‐FCl‐2, and BTP‐FCl‐FCl‐3 respectively.

**Figure** **5**a–d, Figures S7 and S8, Supporting Information, show the ESP distribution and averaged ESP values of the atoms for BTP‐2F‐2Cl and the three different isomers of BTP‐FCl‐FCl molecules. Although F and Cl atoms show negative values due to their strong electronic negative atoms, the most of the molecules’ surfaces have positive ESP values. As expected, the ESP values of most of the surface and atoms of BTP‐2F‐2Cl and BTP‐FCl‐FCl are the same except the eight closest carbon atoms around the halogen atoms (Figure 5e–g). All the eight closest carbon atoms have positive values (Figure S7, Supporting Information) in all the molecules and the average ESP values and standard deviation of them are summarized in Table S11, Supporting Information. The average value all of the BTP‐FCl‐FCl are a bit larger than that of BTP‐2F‐2Cl, while the standard deviation of the BTP‐FCl‐FCl are smaller (≈25 meV). Larger average ESP resulting stronger IEF due to the larger ESP difference between D/A interface. Smaller standard deviation means lower ESP distribution disorder. Meanwhile, the ESP of the IC‐2F and IC‐2Cl end groups of the BTP‐2F‐2Cl molecule are 91.44 and 73.43 kcal mol^−1^, while the ESP of the end groups on both sides of BTP‐FCl‐FCl are almost equal (Table S12, Supporting Information). Therefore, the IEF of the BTP‐FCl‐FCl/BTR‐Cl interfaces are larger and more uniform than that of BTP‐2F‐2Cl/BTR‐Cl interfaces, which is favorable for exciton dissociation. Moreover, the total dipole moments of BTP‐FCl‐FCl (≈0.4 Debye) are lower than that of BTP‐2F‐2Cl (0.73 Debye) (Table S13, Supporting Information). Considering the complexity of the contact at the D/A interface and worse uniformity of BTR‐Cl:BTP‐2F‐2Cl blend film (Figure 3a,c and Figure 4c,d), the diversity of the orientation of BTP‐2F‐2Cl molecules with a bit greater dipole may introduce larger electrostatic disorder at the interface which will scatter the moving charges, and thereby reducing the performance of devices.^[^ ^46^, ^47^ ^]^ In short, the suitable ESP and smaller dipole of BTP‐FCl‐FCl promote the photo‐induced electrons transfer from donor to acceptor, thereby increasing the PCE, which is consistent with the experimental observations.

## 3 Conclusion

In summary, single‐junction ASM OSCs with PCEs of 15.3% have been demonstrated by a locally asymmetric electron acceptor BTP‐FCl‐FCl and BTR‐Cl donor. Compared with the globally asymmetric BTP‐2F‐2Cl based devices, the BTP‐FCl‐FCl based devices show faster charge extraction, longer charge carrier lifetime, and additionally, much lower recombination. Therefore, optimized FF and *J*_SC_ are improved from 71.41% to 75.36% and 22.35 to 24.58 mA cm^−2^, leading to a higher PCE of 15.3%. Our calculation show that BTP‐FCl‐FCl has lower total dipole moment, larger average ESP, and lower distribution disorder of the eight closest carbon atoms around the halogen atoms than that of the BTP‐2F‐2Cl. The intermolecular electric fields between the BTP‐FCl‐FCl/BTR‐Cl interfaces are larger and more uniform than that of BTP‐2F‐2Cl/BTR‐Cl interface, which is beneficial for charges separation, extraction and migration, thereby increasing the PCE. The trap‐assistant SRH recombination of BTP‐FCl‐FCl based device is reduced which led to a prolonged carrier lifetime. Moreover, the lower total dipole moment and lower ESP distribution disorder of BTP‐FCl‐FCl provides suitable crystallinity contributing to more uniform charge separation networks and a significantly improved FF. Our findings demonstrated that this locally asymmetric structure strategy paves a new avenue to further enhance high‐performance ASM OSCs.

## 4 Experimental Section

The detailed materials synthesis and characterization, device fabrication, and characterization can be found in the Supporting Information.

##### Statistical Analysis

Statistical analyses were performed using Origin 9.0 software. Data were reported as the mean ± SD. Sample size for each statistical analysis is described in the corresponding table.

## Conflict of interest

The authors declare no conflict of interest.

## Supporting information

Supporting Information
