# Supplementary material for: 15.3% Efficiency All‐Small‐Molecule Organic Solar Cells Achieved by a Locally Asymmetric F, Cl Disubstitution Strategy
Source: Adv Sci (Weinh). 2021 Feb 22;8(8):2004262. doi: 10.1002/advs.202004262 (PMC8061398; doi:10.1002/advs.202004262)
Supplement: Supplementary file 1 — Supporting Information [file ADVS-8-2004262-s001.pdf]

© 2020 Wiley-VCH GmbH

ADVANCED SCIENCE
Open Access

Supporting Information

for *Adv. Sci.*, DOI: 10.1002/advs.202004262

15.3% Efficiency All-Small-Molecule Organic Solar Cells Achieved by a Locally Asymmetric F, Cl Disubstitution Strategy

Dingqin Hu[a, c] #, Qianguang Yang[a, b] #, Yujie Zheng[c] #, Hua Tang[a, b], Sein Chung[d], Ranbir Singh[e], Jie Lv[a], Jiehao Fu[a], Zhipeng Kan [a, b], Bo Qin[c], Qianqian Chen[a, c], Zhihui Liao[a, b], Haiyan Chen[a, c], Zeyun Xiao [a, b]*, Kuan Sun [c]* and Shirong Lu [a, b]*

Support information

15.3% Efficiency All-Small-Molecule Organic Solar Cells Achieved by a Locally Asymmetric F, Cl Disubstitution Strategy

Dingqin Hu[a, c] #, Qianguang Yang[a, b] #, Yujie Zheng[c] #, Hua Tang[a, b], Sein Chung[d], Ranbir Singh[e], Jie Lv[a], Jiehao Fu[a], Zhipeng Kan [a, b], Bo Qin[c], Qianqian Chen[a, c], Zhihui Liao[a, b], Haiyan Chen[a, c], Zeyun Xiao [a, b]*, Kuan Sun [c]* and Shirong Lu [a, b]*

[a] Chongqing Institute of Green and Intelligent Technology, Chongqing School, University of Chinese Academy of Sciences (UCAS Chongqing), Chinese Academy of Sciences, Chongqing, 400714, China

[b] Chongqing School, University of Chinese Academy of Sciences, Chongqing, 400714, China

[c] Key Laboratory of Low-Grade Energy Utilization Technologies and Systems (Ministry of Education), School of Power Engineering, Chongqing University, Chongqing 400044, P.R. China

[d] Department of Chemical Engineering Pohang University of Science and Technology Pohang 790-784, South Korea

[e] Department of Energy & Materials Engineering, Dongguk University, Seoul, 100-715, Republic of Korea

# The authors contribute equally.

E-mail: xiao.z@cigit.ac.cn; kuan.sun@cqu.edu.cn; lushirong@cigit.ac.cn;

Experimental section

1. Materials

All reagents and solvents, unless otherwise specified, were purchased from Energy Chemical, Tansoole, Suna Tech, Aldrich and JiangSu GE-Chem Biotech., Ltd. and were used without further purification. Phen-NaDPO was purchased from 1 materials. The PEDOT:PSS (Bayer Baytron 4083) was purchased from Heraeus. BTR-Cl, BTP-2F-2Cl (SY2) and BTP-FCl-FCl were synthetized following previous reports.

Synthesis of BTP-FCl-FCl. Compound BT-CHO (102.7 mg, 0.1 mmol), compound IC-FCl (147.9 mg, 0.6 mmol) were dissolved in dry $CHCl_3$ (20 mL). Then, 0.5 mL of pyridine was added slowly. The reaction was stirred for 4 hours at room temperature. Then the solution was poured into methanol and the precipitate was filtered off and washed with methanol. The mixture was purified by silica gel column chromatography using chloroform as the eluent. 141.9 mg of black solid was obtained as the product (yield 95.62%). ^{1}H NMR (400 MHz, $CDCl_3$) δ 9.15 (d, 2H), 8.77 (d, 0.59H), 8.48 (d, 1.41H), 7.98 (d, 1.41H), 7.68 (d, 0.59H), 4.90 – 4.71 (m, 4H), 3.23 (q, 4H), 2.15 (q, 2H), 1.89 (p, 4H), 1.51 (q, 4H), 1.43 – 1.18 (m, 32H), 1.07 (q, 12H), 0.93 – 0.84 (m, 6H), 0.79 (qd, 6H), 0.70 (tt, 6H). ^{13}C NMR (101 MHz, $CDCl_3$) δ 186.04, 160.41, 158.64, 153.97, 147.48, 145.23, 137.80, 136.19, 136.11, 135.36, 133.88, 133.55, 133.42, 133.36, 130.70, 128.44, 128.24, 125.81, 124.78, 119.86, 115.00, 114.50, 113.68, 77.34, 77.03, 76.71, 68.83, 55.72, 40.41, 31.92, 31.20, 30.32, 29.83, 29.71, 29.65, 29.63, 29.52, 29.45, 29.38, 29.35, 27.69, 23.29, 22.86, 22.69, 14.13, 13.77, 10.30, 0.00. ^{19}F NMR (376 MHz, CDCl3) δ -101.04, -101.06, -101.09, -102.18, -102.20, -102.22. MS (MALDI) m/z calcd. for $C_{82}H_{86}F_2N_8O_2S_5$ $[M]^+$: 1484.8408, found 1484.4878.

2. Material Characterizations

(a) **^{1}H NMR** (400 MHz), **^{13}CNMR** (100 MHz) and **^{19}F NMR** spectra were measured on a Bruker AVANCE III HD 400MHz/600 MHz spectrometers. Mass spectra were recorded on a Shimadzu spectrometer. Electrochemical measurements were performed with a CIH660e electrochemical workstation, using a glassy carbon button electrode as the working electrode, a platinum wire as the auxiliary electrode, and a Ag/Ag^{+} glass electrode as the reference electrode. The Ag/Ag^{+} reference electrode was calibrated using the ferrocene/ferrocenium (Fc/Fc^{+}) redox couple. Fc/Fc^{+} is taken to be 4.8 eV relative to the vacuum level.

(b) Thermogravimetric Analyzer **(TGA)** was carried out on a WCT-2 thermal balance under protection of nitrogen at a heating rate of 10 ℃/min.

Thin Film Characterizations

(a) Ultraviolet−visible light (**UV-vis**) absorbance spectra were recorded on a Perkin Eimer Lambda 365 spectrophotometer.

(b) Topographic images of the films were obtained from a Bruker atomic force microscopy (**AFM**) with the type of dimension edge with Scan AsystTM in the tapping mode using an etched silicon cantilever at a nominal load of ~2nN, and the scanning rate for a 2 μm×2 μm image size was 1.5 Hz.

(c) Transmission electron microscope (**TEM**) studies were conducted with a FEI Tecnai G2 F20 electron microscopy to investigate the phase distribution of the active layer，and with the scale bar is 200 nm.

(d) Two-dimensional grazing incident wide angle X-ray scattering (2D-GIWAXS) measurements were carried out at the PLS-II 3C beam line of Pohang Accelerator Laboratory, Korea.

3. Device Fabrication and characterizations

The device structures were ITO/PEDOT:PSS/Active layer/Phen-NaDPO/Ag. ITO coated glass substrates were cleaned with detergent water, deionized water, acetone and isopropyl alcohol in an ultrasonic bath sequentially for 30 min, and further treated with UV exposure for 15 min in a UV-ozone chamber. A thin layer (ca. 30 nm) of PEDOT:PSS (Bayer Baytron 4083) was first spin-coated on the substrates with 4000 rpm and baked at 120 °C for 10 min under ambient conditions. The substrates were then transferred into a nitrogen-filled glove box. For BTR-Cl:acceptor-based OSCs, the optimized concentration was 20 mg/ml chloroform solution with D:A ratio of 1.8:1 (w/w) and the thickness is about 110 nm. After spin coating, the blend films were treated with chlorobenzene solvent for 30 s and annealed at 120 °C for 5 mins. Then Phen-NaDPO as the electron transporting layer was spin-coated on the active layer by 2000 rpm from isopropyl alcohol solution. Finally, the substrates were transferred to a thermal evaporator, and top electrode was evaporated at a pressure of 2×10^{-5} Pa.

The external quantum efficiency (*EQE*) was performed using certified IPCE equipment (Enli Technology Co., Zolix Instruments, Inc, Solar Cell Scan 100). The *J–V* curves were measured under AM 1.5 G (100 mW cm^{-2}) (Enli Technology Co., Ltd. SS-X50R). The *J–V* measurement signals were recorded by a Keithley 2400 source-measure unit. Device area of each cell was 0.1 cm^2.

4. Carrier Mobility Measurements

The carrier mobility (hole and electron) of photoactive active layer was obtained by fitting the dark current of hole/electron-only diodes to the space-charge-limited current (SCLC) model. Hole-only diode configuration: Glass/ITO/PEDOT:PSS/active layer/MoO_3/Ag; Here, V_{bi}=0 V (flat band pattern formed by PEDOT:PSS-MoO_3). Electron-only diode configuration:

Glass/ITO/ZnO/Phen-NaDPO/Active layer/Phen-NaDPO/Ag; Here, V_{bi}=0.5V was used following the protocol reported. The active layer thickness was determined by a Tencor surface profilometer. The electric-field dependent SCLC mobility was estimated using the following equation:

$$J(V) = \frac{9}{8}\varepsilon_0\varepsilon_r\mu_0 exp\left(0.89\beta\sqrt{\frac{V-V_{bi}}{L}}\right)\frac{(V-V_{bi})^2}{L^3}$$

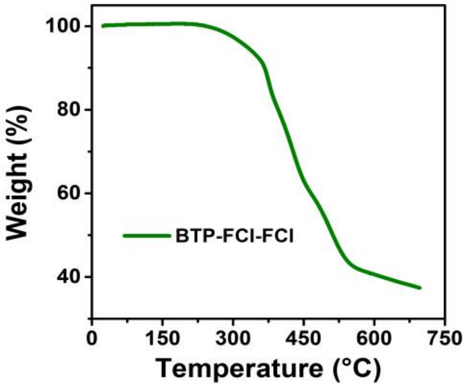

Figure S1. Thermogravimetric analysis (TGA) of BTP-FCl-FCl. The material shows high thermal-stability under nitrogen atmosphere with a heating rate of 10 ℃/min.

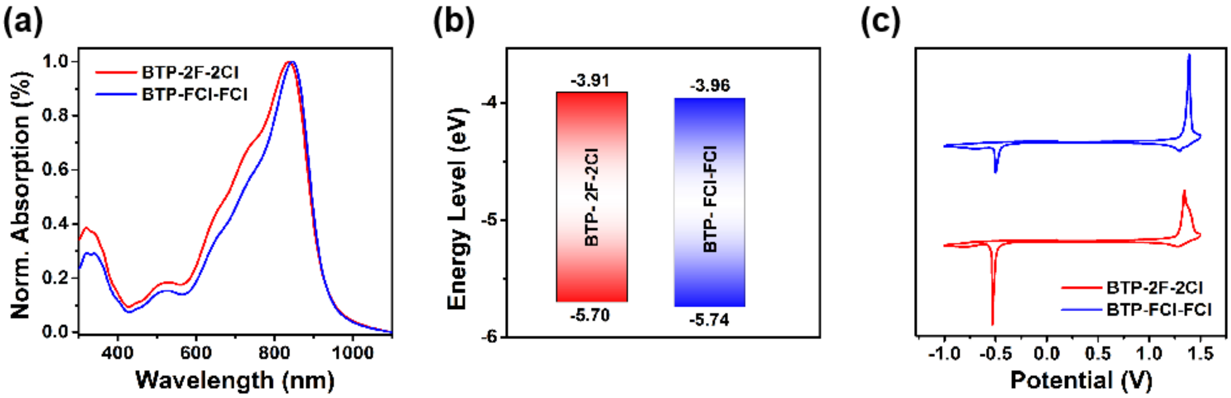

Figure S2. **(a)** Normalized UV-vis spectra, **(b)** Energy Level, **(c)** Cyclic Voltammogram of BTP-2F-2Cl, BTP-FCl-FCl.

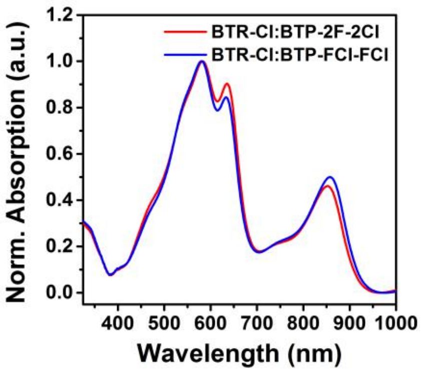

Figure S3. Absorption of BTR-Cl:acceptor based organic solar cells.

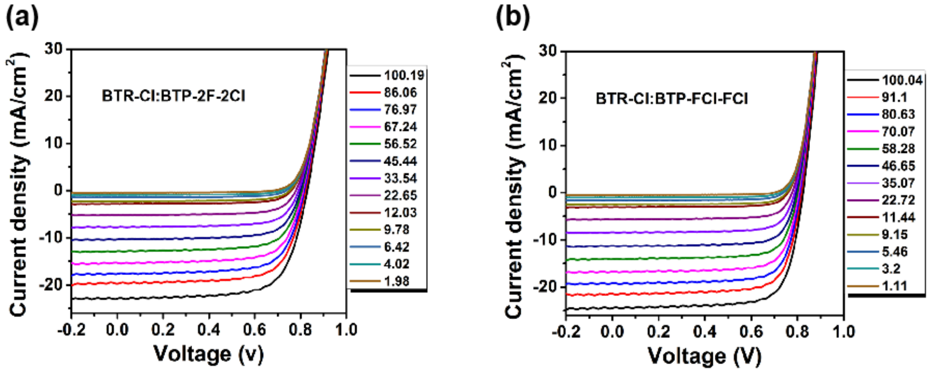

Figure S4. *J*-*V* curves of (a) BTR-Cl:BTP-2F-2Cl, (b) BTR-Cl:BTP-FCl-FCl based organic solar cells under different light intensities.

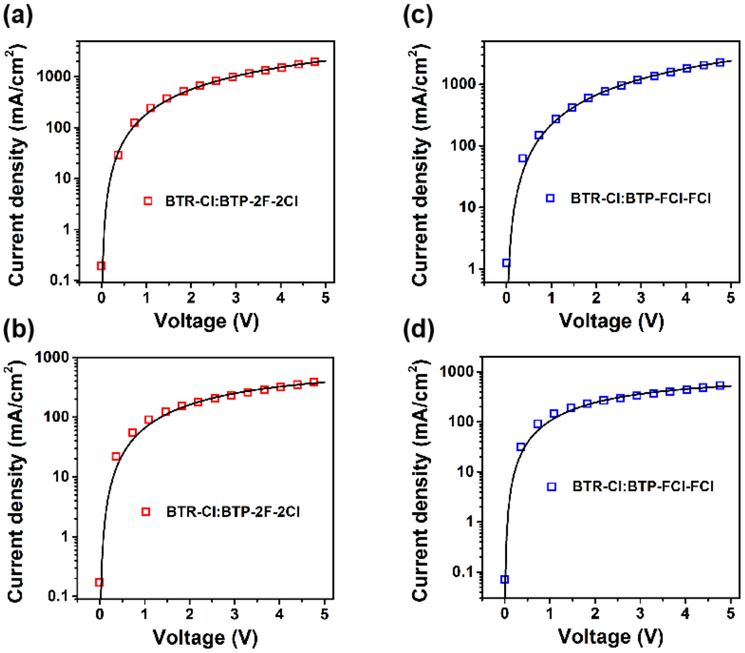

Figure S5. (a), (b) electron and hole mobility fitting curves of BTR-Cl:BTP-2F-2Cl; (c), (d) electron and hole mobility fitting curves of BTR-Cl:BTP-FCl- FCl based organic solar cells.

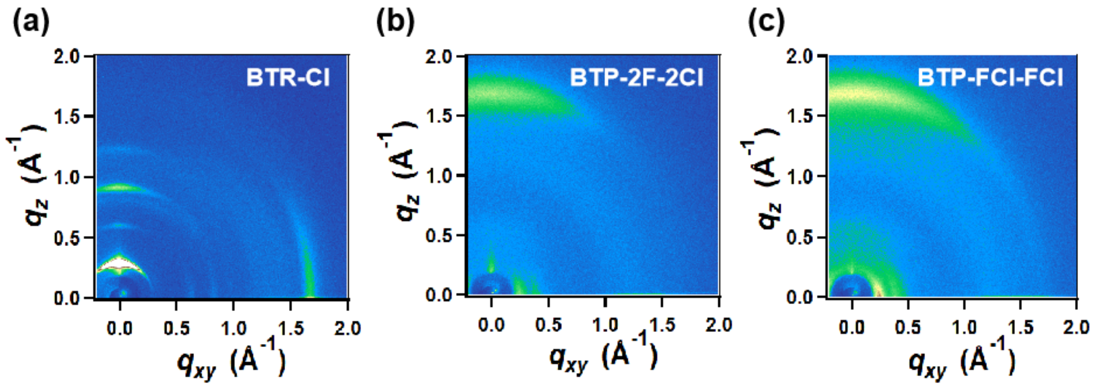

Figure S6. (a)~(c) 2D GIWAXS patterns of BTR-Cl, BTP-2F-2Cl and BTP-FCl-FCl pure films.

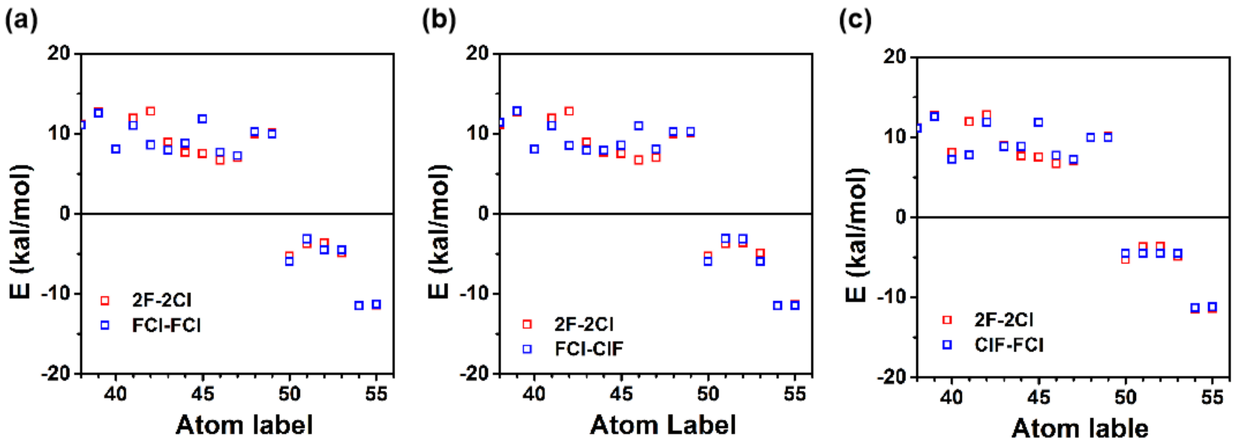

Figure S7. (a)~(c) Averaged ESP values of the atoms around the halogen atoms of the BTP-2F-2Cl compared with BTP-FCl-FCl-1, BTP-FCl-FCl-2 and BTP-FCl-FCl-3, respectively.

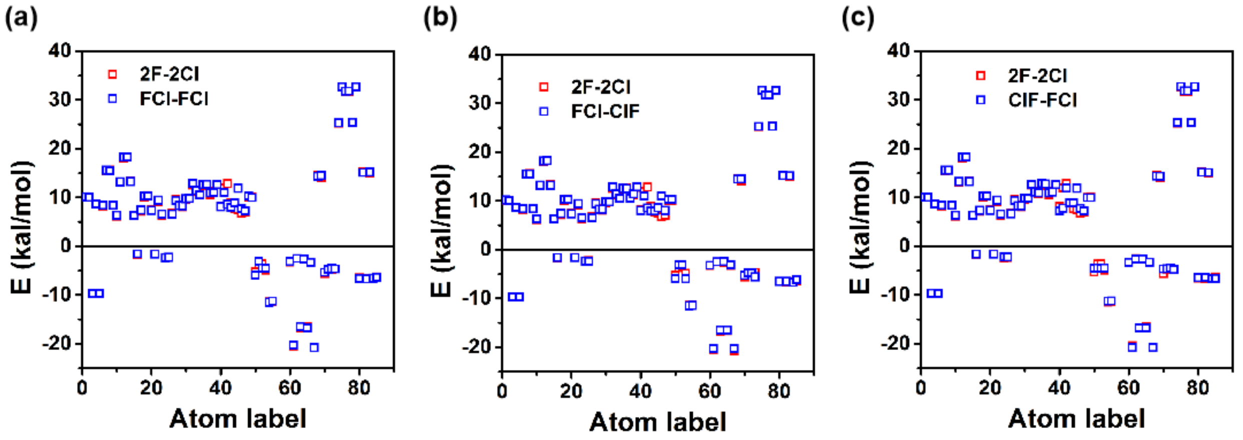

Figure S8. (a)~(c) Averaged ESP values of the atoms in the conjugated backbones for BTP-2F-2Cl compared with BTP-FCl-FCl-1, BTP-FCl-FCl-2 and BTP-FCl-FCl-3, respectively.

Table S1. Summary of optical properties, electronic energy level of BTP-2F-2Cl and BTP-FCl-FCl.

Material	λ_{max}^{sol} [nm]	λ_{max}^{film}[nm]	λ_{onset}^{film} [nm]	E_g^{opt} [eV]	HOMO^{CV} [eV]	LUMO^{CV} [eV]
BTP-2F-2Cl	738.1	835.5	934.7	1.33	-5.70	-3.91
BTP-FCl-FCl	740.6	845.5	933.7	1.33	-5.74	-3.96

[a] Calculated from the onset absorption of thin films.

[b] $E_g^{opt} = 1240/\lambda_{onset}^{film}$ (eV).

Table S2. Photovoltaic parameters of the BTR-Cl:BTP-FCl-FCl based solar cells with different thickness of active layer.

**Thickness (nm)**	V_{OC} **(mV)**	J_{SC} **(mA/cm^2)**	***FF*** **(%)**	[a)]***PCE*** **(%)**
90	796.3	22.38	64.83	11.55 (11.10±0.16)
110	807.1	23.19	73.36	13.73 (13.55±0.14)
120	805.3	23.37	74.15	13.95 (13.67±0.17)
130	804.2	22.64	71.36	12.99 (12.74±0.21)
180	803.0	21.85	65.52	11.50 (11.29±0.13)

a) Statistical data obtained from at least 15 devices.

Table S3. Photovoltaic parameters of the BTR-Cl:BTP-FCl-FCl based solar cells with different TA temperature.

**Device Condition**	V_{OC} **(mV)**	J_{SC} **(mA/cm^2)**	***FF*** **(%)**	[a)]***PCE*** **(%)**
TA=110℃/5 m	848.2	22.48	59.97	11.44 (11.13±0.29)
TA=120℃/5 m	839.6	23.76	68.91	13.75 (13.59±0.16)
TA=130℃/5 m	823.9	23.18	69.72	13.32 (13.19±0.19)
TA=140℃/5 m	816.6	22.90	70.90	13.26 (13.11±0.10)

a) Statistical data obtained from at least 15 devices.

Table S4. Photovoltaic parameters of the BTR-Cl:BTP-FCl-FCl based solar cells with different CF SVA times.

Device Condition	V_{OC} (mV)	J_{SC} (mA/cm^2)	*FF* (%)	$^{a)}$*PCE* (%)
SVA=CF/15 s	843.4	23.03	67.34	13.08 (12.68±0.30)
SVA=CF/25 s	824.5	23.40	72.36	13.96 (13.81±0.19)
SVA=CF/30 s	817.5	23.29	72.40	13.78 (13.53±0.18)

a) Statistical data obtained from at least 15 devices.

Table S5. Photovoltaic parameters of the BTR-Cl:BTP-FCl-FCl based solar cells with different CB SVA times.

Device Condition	V_{OC} (mV)	J_{SC} (mA/cm^2)	*FF* (%)	$^{a)}$*PCE* (%)
SVA=CB/15 s	885.4	16.19	36.32	5.21 (5.06±0.13)
SVA=CB/20 s	869.6	18.37	48.75	7.79 (7.59±0.19)
SVA=CB/30 s	852.7	22.91	65.27	12.75 (12.61±0.15)
SVA=CB/40 s	846.0	23.51	70.17	13.96 (13.88±0.08)
SVA=CB/50 s	837.1	23.73	71.78	14.26 (14.11±0.12)
SVA=CB/60 s	828.9	23.46	72.79	14.15 (14.02±0.10)
SVA=CB/80 s	818.9	22.71	73.02	13.58 (13.19±0.21)

a) Statistical data obtained from at least 15 devices.

Table S6. Photovoltaic parameters of the BTR-Cl:BTP-FCl-FCl based solar cells with different ratio of D/A.

Device Condition	V_{OC} (mV)	J_{SC} (mA/cm^2)	*FF* (%)	$^{a)}$*PCE* (%)
D/A=1.6:1	802.6	23.26	64.83	13.30 (13.15±0.10)
D/A=1.8:1	811.0	23.35	73.36	14.28 (14.11±0.12)
D/A=2:1	800.5	22.22	71.36	13.38 (13.19±0.13)

a) Statistical data obtained from at least 15 devices.

Table S7. Photovoltaic parameters of the BTR-Cl:BTP-FCl-FCl based device with different treatment.

**Device Condition**	**V_{OC} (mV)**	**J_{SC} (mA/cm^2)**	***FF* (%)**	**[a]*PCE* (%)**
SVA CF/30s +120℃/5 m	814.1	23.15	76.53	14.43 (14.25±0.09)
SVA CB/30s +120℃/5 m	825.4	24.58	75.36	15.29 (15.14±0.09)
SVA CB/40s +120℃/5 m	818.6	24.39	74.59	14.89 (14.56±0.24)
SVA CB/50s +120℃/5 m	812.6	23.91	74.25	14.43 (14.21±0.11)

a) Statistical data obtained from at least 15 devices.

Table S8. Mobility values of BTP-FCl-FCl and BTP-2F-2Cl based devices.

**Device Condition**	**μ_e (×10^{-3} cm^{2}V^{-1}s^{-1})**	**μ_h (×10^{-3} cm^{2}V^{-1}s^{-1})**	**μ_e /μ_h**
BTR-Cl:BTP-2F-2Cl	1.22 (0.98 ± 0.21)	1.03 (0.87 ± 0.14)	1.18 (1.11 ± 0.02)
BTR-Cl:BTP-FCl-FCl	2.21 (2.03 ± 0.17)	2.02 (1.88 ± 0.12)	1.09 (1.07 ± 0.02)

Table S9. Exciton dissociation and charge collection efficiency parameters of BTP-FCl-FCl and BTP-2F-2Cl based devices.

**Device Condition**	**J_{sc} (mA/cm^2)**	**J_{sat} (mA/cm^2)**	**J_{max} (mA/cm^2)**	**[a]η_{diss} (%)**	**[b]η_{coll} (%)**
BTR-Cl:BTP-2F-2Cl	23.05	24.19	20.21	95.29	83.55
BTR-Cl:BTP-FCl-FCl	24.80	25.56	21.89	97.02	88.26

a) $\eta_{diss}=J_{sc}/J_{sat}$, b) $\eta_{coll}=J_{max\,power}/J_{sat}$.

Table S10. Analysis data of the two-dimensional GIWAXS results in out-of-plane direction (OOP) of BTP-FCl-FCl and BTP-2F-2Cl based blend films.

Blend film	Lattice plane	Peak location (A^{-1})	d-spacing (Å)	Coherence length (Å)
		q_z	q_z	q_z
	010	1.674	3.75	48.07
	010	1.669	3.76	45.03

Table S11. Average ESP values and standard deviation of the 8 closest carbon atoms around the halogen atoms.

-	BTP-2F-2Cl	BTP-FCl-FCl	BTP-FCl-ClF	BTP-ClF-FCl
Average ESP (meV)	384.26	387.11	386.09	387.91
Standard deviation (meV)	92.93	66.66	54.05	77.70

Table S12. The total ESP values of the end group of BTP-2F-2Cl and BTP-FCl-FCl molecules. The total ESP are obtained by sum the average ESP values of all the atoms of the end groups.

Molecule<br>ESP (kcal/mol)	Left side	Right side	Core	Total
BTP-2F-2Cl	91.45	73.43	668.67	833.55
BTP-FCl-ClF	83.96	83.95	667.11	835.02
BTP-ClF-FCl	82.56	82.44	669.85	834.85
BTP-FCl-FCl	84.32	82.30	668.31	834.93

Table S13. Summarize the total energy and dipole moment of the molecules.

-	BTP-2F-2Cl	BTP-FCl-FCl	BTP-FCl-ClF	BTP-ClF-FCl
Total energy (Hartree)	-5539.316169	-5539.320572	-5539.321165	-5539.319978
Dipole moment (Debye)	0.7288	0.3964	0.2335	0.4973

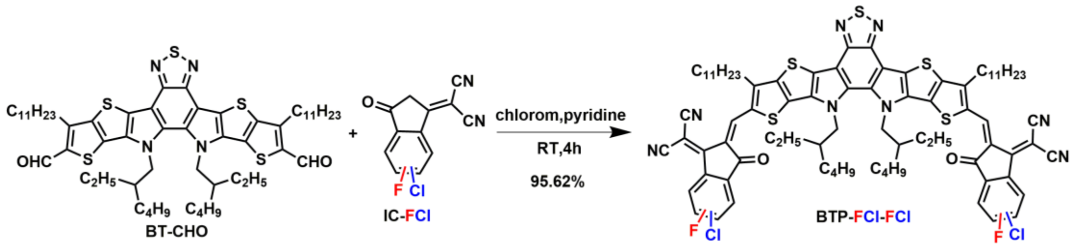

Scheme S1. Synthetic route of BTP-FCl-FCl.

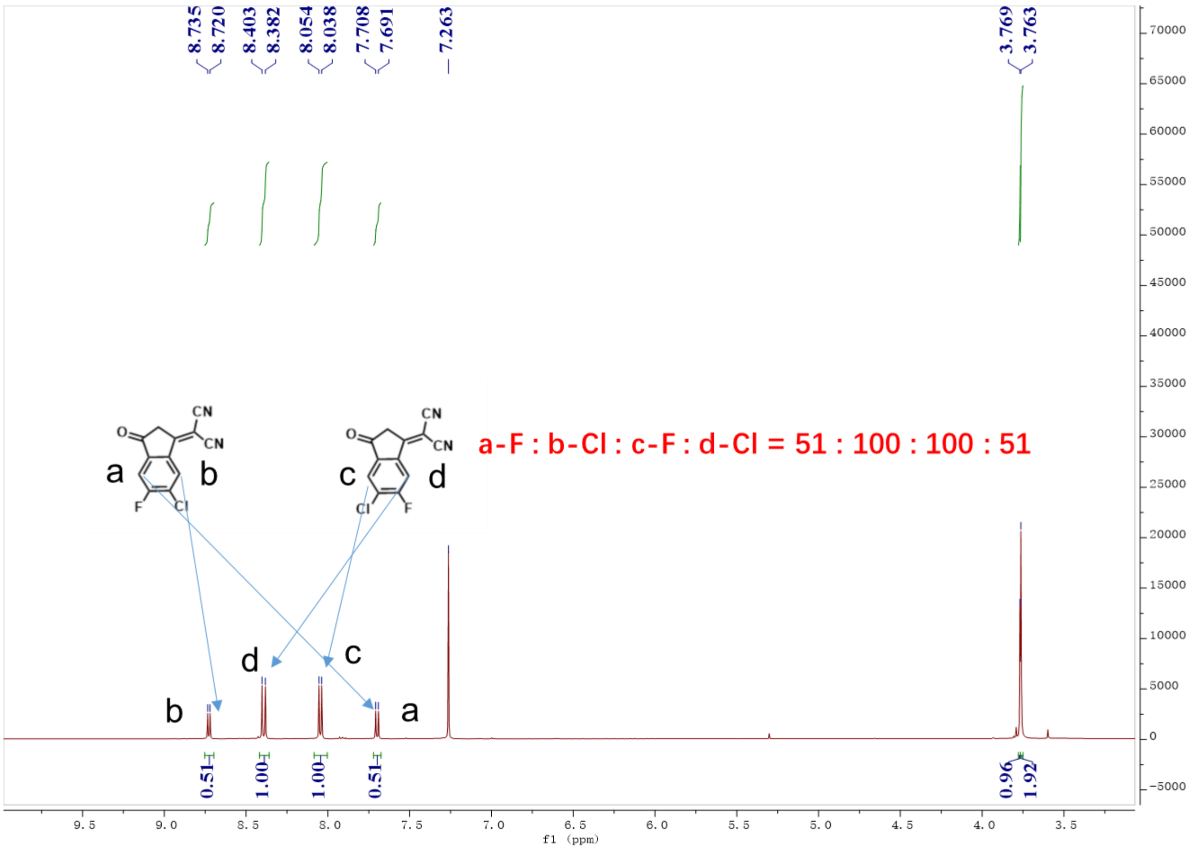

Figure S7. ^{1}H NMR spectrum of **IC-FCl** in $CDCl_3$.

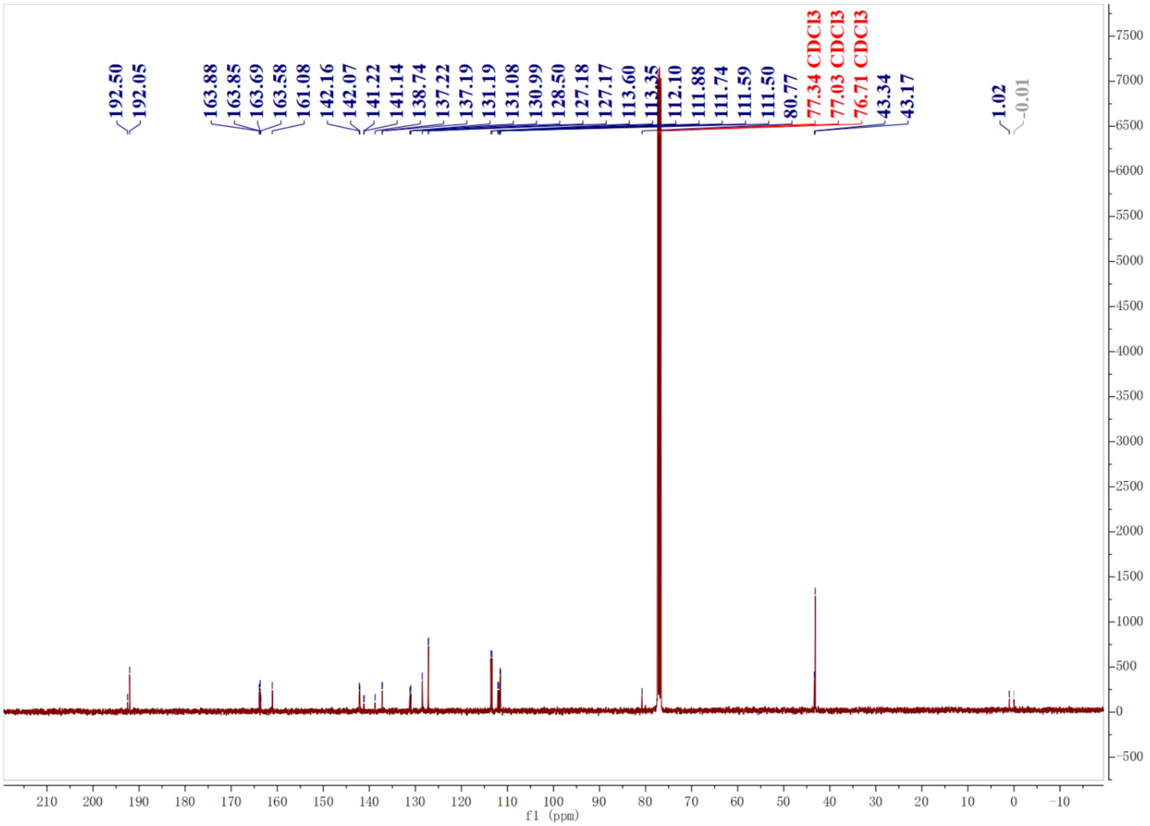

Figure S8. ^{13}C NMR spectrum of **IC-FCl** in $CDCl_3$.

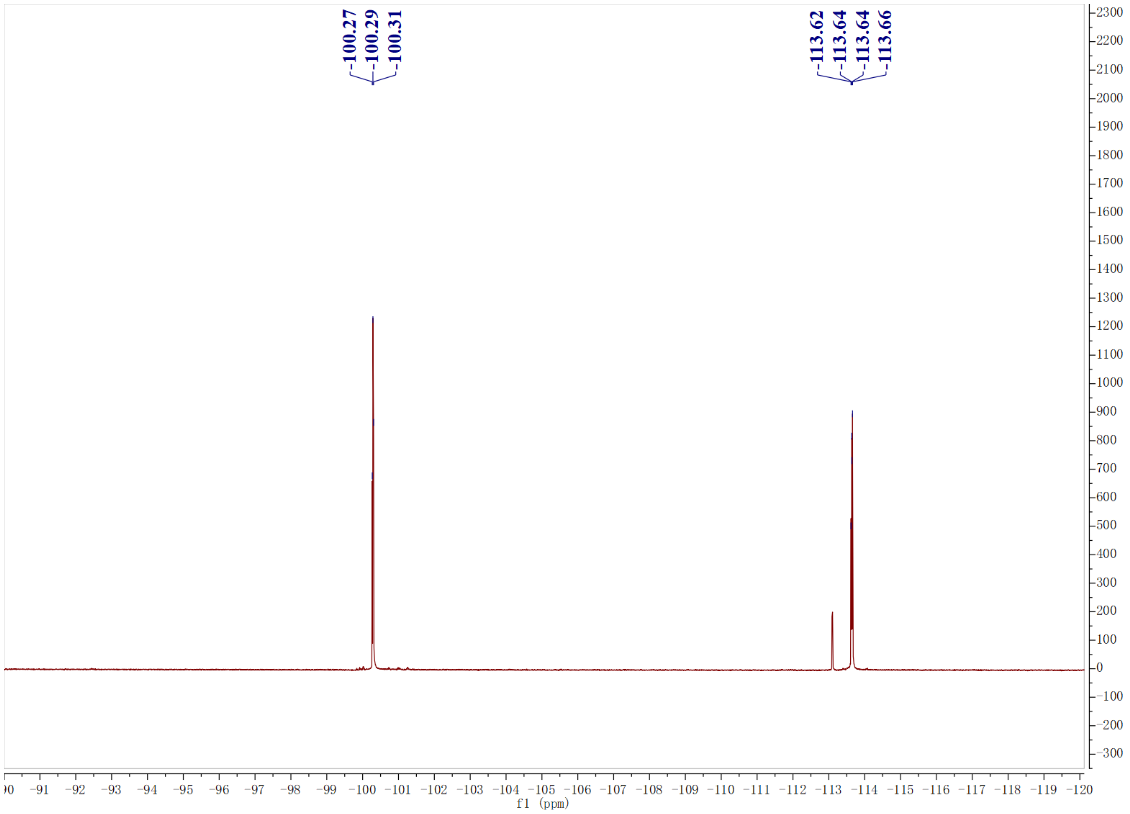

Figure S9. ^{19}F NMR spectrum of **IC-FCl** in $CDCl_3$.

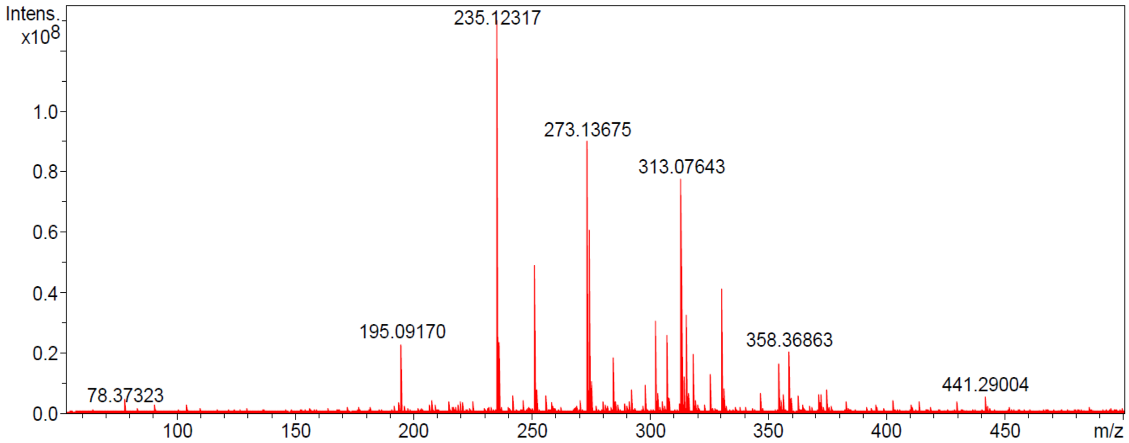

Figure S10. MS (MALDI) spectrum of **IC-FCl.**

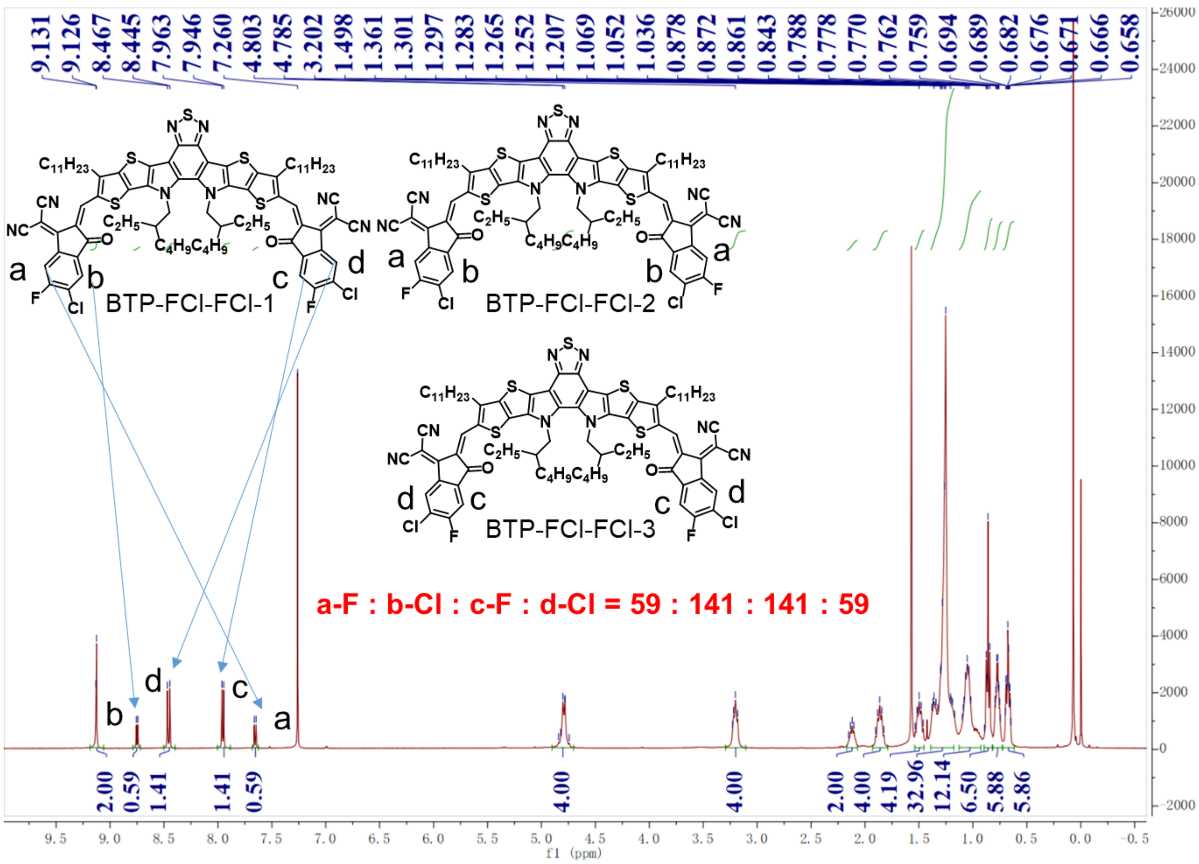

Figure S11. ^{1}H NMR spectrum of **BTP-FCl-FCl** in $CDCl_3$.

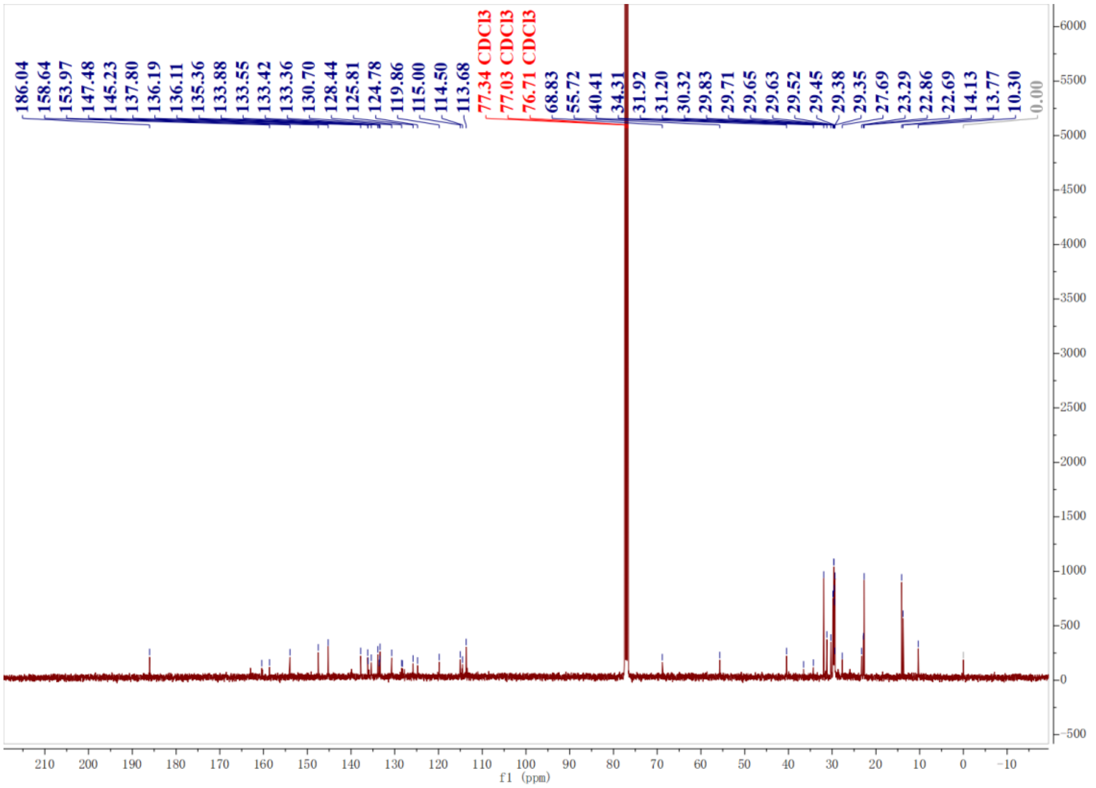

Figure S12. ^{13}C NMR spectrum of **BTP-FCl-FCl** in $CDCl_3$.

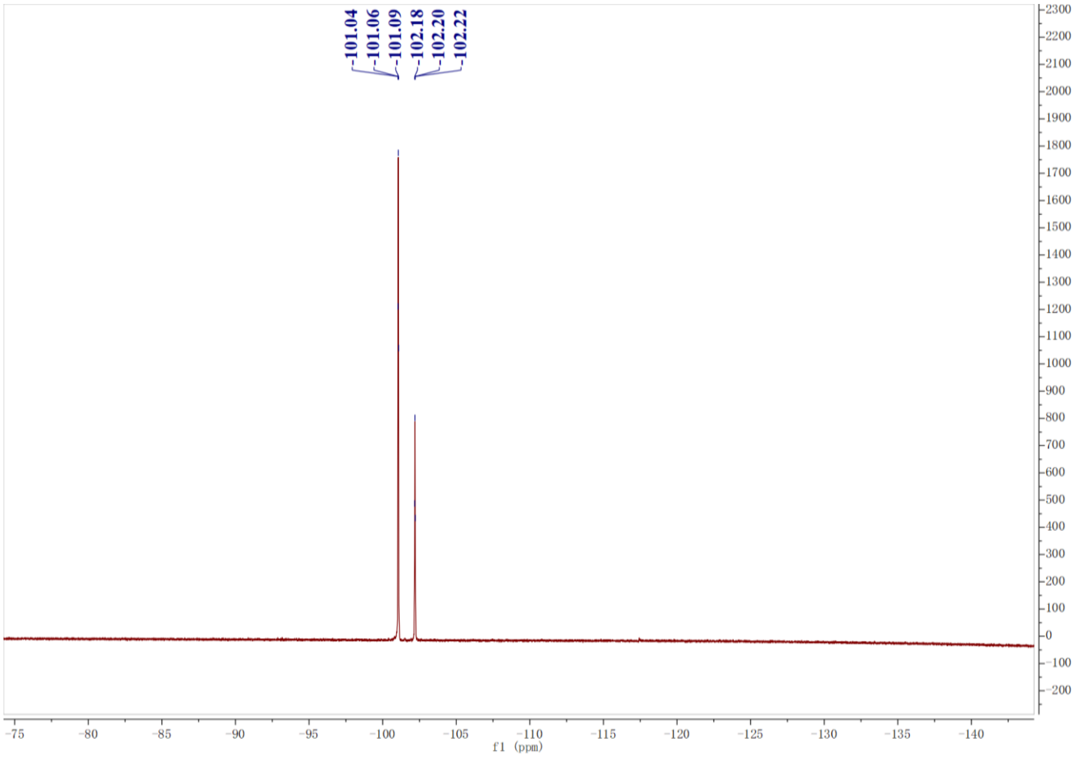

Figure S13. ^{19}F NMR spectrum of **BTP-FCl-FCl** in $CDCl_3$.

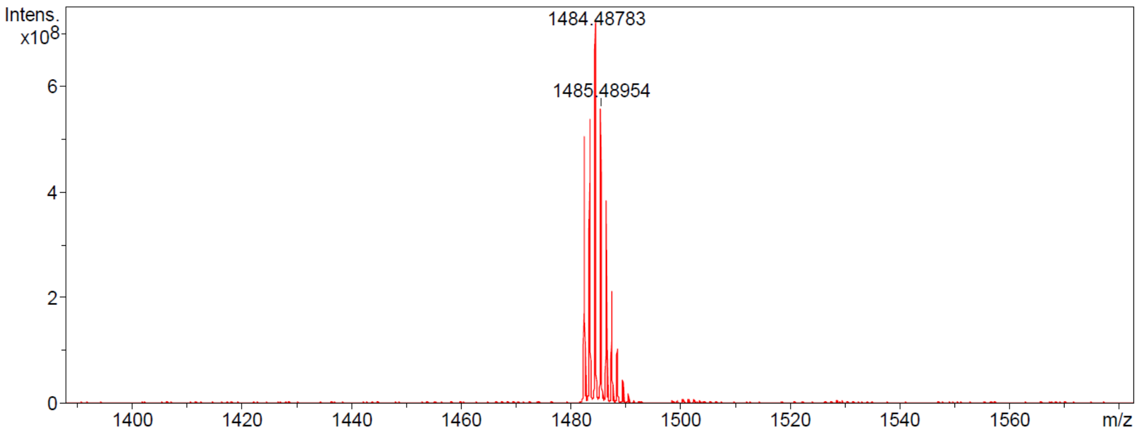

Figure S14. MS (MALDI) spectrum of **BTP-FCl-FCl.**
